# Gloss and Surface Roughness of Stained and Polished 3D-Printed Resins Used for the Fabrication of Hybrid Denture Prosthesis: An In Vitro Study

**DOI:** 10.3390/jfb17100480

**Published:** 2026-09-22

**Authors:** Sarah M. Alnafaiy, Nawaf Labban, Alhanoof Aldegheishem, Refal Albaijan, Saad S. AlResayes, Mohammed Aldhafyan

**Affiliations:** 1 Department of Clinical Dental Sciences, College of Dentistry, Princess Nourah bint Abdulrahman University, P.O. Box 84428, Riyadh 11671, Saudi Arabia; 2 Department of Prosthetic Dental Sciences, College of Dentistry, King Saud University, P.O. Box 60169, Riyadh 11545, Saudi Arabia; 3 Department of Prosthodontics, College of Dentistry, Prince Sattam bin Abdulaziz University, P.O. Box 173, Al-Kharj 11942, Saudi Arabia; 4 Restorative Dental Sciences Department, College of Dentistry, King Saud University, P.O. Box 60169, Riyadh 11545, Saudi Arabia; maldhafyan@ksu.edu.sa

**Keywords:** 3D-printing, CAD/CAM, hybrid denture, gloss, resin composite, surface roughness

## Abstract

Despite the increasing use of additively manufactured restorative materials, comparative information regarding their surface gloss and roughness after different surface treatments and simulated oral challenges remains limited. This study evaluated the gloss and surface roughness of stained and polished 3D-printed resins subjected to acidic storage followed by simulated tooth brushing. Two 3D-printed resins (ONX and Tough 2 [T2]) were compared with a milled material (Lava Ultimate [LU]), which was used as a control. A total of 120 specimens (40 per material) were fabricated, surface-treated (stained or polished), and randomly categorized into acid or water storage/tooth brushing. A gloss meter and non-contact profilometer were used to measure the gloss and surface roughness of the materials. Data analysis was performed using one-way ANOVA, Welch’s pairwise *t*-tests with Holm adjustment, and HC3-robust Type III factorial models, with significance set at α = 0.05. Gloss significantly decreased, whereas surface roughness (Ra) significantly increased after storage/brushing across the experimental groups (*p* < 0.05), except for T2 subjected to acid storage/brushing, which showed no significant change in R_a_ (*p* = 0.11). Both gloss and R_a_ were significantly affected by the material type (*p* < 0.001). For gloss, significant effects of material and surface treatment were observed (*p* < 0.001). For R_a_, significant effects of material, surface treatment, and storage/brushing were detected (*p* < 0.001). Material type and surface treatment significantly influenced gloss, whereas storage/brushing effect had no significant effect. In contrast, material type, surface treatment, and storage/brushing significantly influenced surface roughness. The milled LU generally exhibited higher gloss and lower roughness than the 3D-printed ONX and T2 materials, particularly among stained specimens, while ONX generally showed more favorable surface properties than T2.

## 1. Introduction

Advances in computer-aided design and computer-aided manufacturing (CAD/CAM), including subtractive milling and additive manufacturing, have significantly advanced dental prosthesis fabrication by improving precision, efficiency, and chairside workflows [1,2]. Removable dental prosthesis fabrication via CAD/CAM milling and 3D printing has proven to be clinically acceptable [3]. Milling and 3D printing are advantageous and unique in their own perspective. Milling is widely recognized for its high dimensional precision, consistent surface quality, and ability to produce dense and mechanically robust components with relatively smooth surfaces and limited post-processing. In contrast, 3D-printing offers greater design flexibility and material efficiency, enabling the fabrication of complex and patient-specific geometries, surgical guides, lattice structures, and increasingly, definitive dental restorations [1,4]. Recent systematic reviews indicate that optimized 3D-printing protocols can achieve clinically acceptable accuracy and, for selected applications, comparable outcomes to milling. However, evidence also suggests that milling may retain an advantage for certain definitive restorations in terms of marginal fit and trueness. Therefore, the quality of a digitally fabricated restoration should be considered a function of the specific material–manufacturing system and processing protocol rather than being attributed solely to the manufacturing approach [5,6].

Hybrid dentures are fixed-removable restorations with semi-precision attachments and were introduced to replace unstable and uncomfortable mandibular dentures [7]. They draw primary support from implants in the jawbone and are built on a robust metal framework. By combining the strengths of fixed restorations with the adaptability of dentures, they offer stable function and durability, compensate for soft-tissue and bone loss, and ultimately maintain superior esthetics [7]. Hybrid dentures offer good retention, stability, and support, which are comparable to those of fixed restorations, while addressing the esthetic and hygiene aspects of removable designs [7].

Resin nanoceramics (RNCs) have gained increased attention as CAD/CAM restorative materials because they combine the favorable esthetic and wear characteristics of ceramic materials with the machinability, resilience, and damage tolerance of resin-based materials [8,9,10]. Their highly filled resin matrix containing ceramic or inorganic fillers provides a balance of mechanical performance, surface properties, and resistance to the oral environment, making them suitable for a variety of indirect restorative applications, including inlays, onlays, overlays, and veneers [9,11,12,13]. Furthermore, they offer increased energy absorption, resistance to catastrophic fracture, and ease of adjustment, polishing, and repair compared to conventional ceramics [14]. Overall, RNCs provide esthetic, biocompatible, and mechanically robust options in restorative dentistry and implant prosthodontics [15].

The CAD/CAM milling of RNCs facilitates accurate and patient-specific fabrication of dental prostheses [16]. More recently, advances in additive manufacturing have led to the development of a new generation of 3D-printed resin-based and nano-ceramic-filled materials intended for definitive and provisional dental prostheses [17]. These materials represent a transition from conventionally CAD/CAM-milled RNCs toward additively manufactured RNCs, developed to meet the mechanical, esthetic, and functional requirements of dental restorative applications [17,18,19]. Among the newer materials, SprintRay OnX has been developed for the fabrication of 3D-printed provisional hybrid dentures [17], whereas OnX Tough 2 has been designed for fixed hybrid denture applications [18]. These materials are intended to expedite CAD/CAM driven fabrication of full-arch prostheses while offering the manufacturing advantages of additive technology. The development of materials such as OnX and OnX Tough 2 therefore provides a new possibility for 3D printing beyond the simple realm of conventional restorations into more challenging hybrid denture applications.

The esthetic success and biological performance of dental restorations are strongly influenced by surface gloss and topography [20]. Gloss is the amount of specular reflection from a surface, and it is generally higher for smoother and more homogeneous surfaces, which are closer to the optical properties of the natural tooth structure [20,21]. Surface roughness (R_a_) refers to a quantitative assessment of surface texture that is typically defined as the average deviation of a surface profile from the mean plane. There is a consensus that surface roughness above 0.2 μm is correlated with increased retention of plaque and bacteria [22,23]. Furthermore, the mechanical properties of the surface can affect the aesthetics of a restoration by reducing its gloss and increasing light scattering [24,25]. Rougher restorative surfaces may create an ideal environment for bacterial growth, bacterial adhesion, and biofilm retention, which can lead to gingivitis and periodontal disease [23,26]. As a result, a smooth, shiny surface is clinically significant for maintaining the esthetics and long-term surface integrity of restorative materials and reducing plaque retention [20].

In addition to the manufacturing method, newer-generation 3D-printed restorative materials also differ from milled RNC in material composition, filler characteristics, polymerization behavior, and surface structure. These differences can affect their resistance to mechanical wear, chemical degradation, and changes in surface topography and optical properties during service [10,27]. In clinical practice, 3D-printed restorations may be subjected to different finishing procedures, including polishing or application of a stain/glaze layer, and these surface treatments may modify their initial gloss and roughness as well as their subsequent resistance to oral challenges. The two relevant clinical mechanisms that can lead to surface degradation of the material are acidic dietary exposure and toothbrushing. The combined influence of surface treatment, chemical exposure, and mechanical toothbrushing on the surface characteristics of modern 3D-printed restorative materials remains poorly understood. In addition, standardized surface-treatment and aging conditions are not available for direct comparison to established milled RNC.

This knowledge gap has a direct impact on clinical relevance, as the maintenance of a smooth and glossy surface after oral challenges can affect decision-making in material selection and finishing procedures. Surface treatment that results in an initial favorable appearance can be affected by significant changes after chemical and mechanical aging, which may reduce the clinical benefit. In contrast, a material and surface treatment combination that has a low surface roughness and a high gloss after simulated oral challenges may offer more consistent long-term surface performance. Therefore, evaluating both the initial and aged surface characteristics is important when determining the suitability of newly introduced 3D-printed materials for definitive or long-term prosthetic applications. This information could assist clinicians in selecting the appropriate materials and surface-treatment procedures while recognizing that in vitro surface behavior does not by itself establish clinical longevity or biological outcomes.

Therefore, this study aimed to assess and compare the gloss and surface roughness of stained and polished 3D-printed materials (Tough 2 and ONX) and a milled Lava Ultimate (LU) subjected to acid or water storage and tooth brushing. The first null hypothesis was that there are no significant main or interactive effects of material type (T2, ONX, and LU), surface treatment (stained and polished), and storage condition (citric acid and distilled water) and tooth brushing on gloss. The second null hypothesis was that there are no significant main or interactive effects of material type (T2, ONX, and LU), surface treatment (stained and polished), and storage condition (citric acid and distilled water) and tooth brushing on surface roughness.

## 2. Materials and Methods

In this study, two 3D-printed resin materials, T2 and ONX, both from the same manufacturer, and one CAD/CAM milling material, Lava Ultimate (LU), used as a control, were tested. Table 1 provides the detailed specifications of each material.

### 2.1. Sample Size and Specimen Preparation

Sample size estimation was based on data reported by Koizumi et al. [28]. The pooled standard deviations were 6.36 for gloss and 0.082 μm for surface roughness (R_a_), with corresponding mean differences of 14.99 gloss units and 0.146 μm, respectively. These values yielded standardized effect sizes (Cohen’s d) of 2.36 for gloss and 1.78 for surface roughness, respectively. Assuming a significance level of α = 0.05 (two-tailed) and a statistical power of 80%, the minimum required sample sizes were calculated as 3 specimens per group for gloss and 5 specimens per group for surface roughness, after rounding upward. Considering the factorial nature of the study (3 materials × 2 surface treatments × 2 storage conditions), the need to evaluate interaction effects, and potential experimental variability or specimen exclusion, 10 specimens were selected for each experimental cell.

A total of 120 rectangular specimens (40 per material), each measuring 12 × 14 × 2.5 mm (±0.05), were prepared from the three study materials. LU specimens were fabricated by sectioning the CAD/CAM blocks to the required thickness using an automated saw (IsoMet 1000 Precision; Buehler, Lake Bluff, IL, USA) with water coolant. For the printed resins, the specimens were initially designed in Rayware (SprintRay, Los Angeles, CA, USA) to the required dimensions (Figure 1a) and then exported as STL (Stereolithography) files, which were sent to a digital light processing (DLP) 3D printer (Pro S, SprintRay, Los Ángeles, CA, USA). The build orientation was set to 0°, and a 50-µm layer thickness was used for ONX, while a 100-µm layer thickness was used for T2 according to the manufacturer’s recommendation. After printing (Figure 1b), each specimen was washed and dried according to the manufacturer’s protocol (ProWash S, SprintRay). Finally, all specimens were post-cured (ProCure 2, SprintRay) using the device’s material-specific, manufacturer-recommended curing times. The prepared specimens from each material were allocated to one of two groups based on surface treatment (stained or polished; *n* = 40).

### 2.2. Specimen Staining

The specimens were stained using the OptiGlaze system (GC International AG, Tokyo, Japan) exactly as directed. Briefly, each surface was abraded with alumina (100 µm) at 2 bar; the specimen was then cleaned with water and dried. A uniformly thin coat of Ceramic Primer II (GC International AG, Luzern, Switzerland) was applied, followed by a single thin layer of OptiGlaze yellow stain, which was light-cured for 40 s [29]. A uniform application was achieved through a standardized protocol to ensure uniform coverage of the entire surface area. Although the glaze thickness was not measured directly, a standardized application protocol was implemented to minimize variation between specimens.

### 2.3. Specimen Polishing

For polishing, each specimen was brought to a uniform finish in stages. First, they were sequentially ground with 400-, 600-, 800-, and 1200-grit silicon carbide papers (LECO Spectrum System, Bloomfield, CT, USA) under continuous water cooling. A low-speed handpiece with medium and fine rubber wheels (green and red; Dedeco International, Long Eddy, NY, USA) was then used at 10,000 rpm. A final pass with diamond polishing paste (MetaDi Supreme, Buehler, Lake Bluff, IL, USA) produced a uniform and glossy surface.

### 2.4. Storage and Simulated Tooth Brushing

From each stained and polished material, the specimens were randomly allocated to two storage conditions (*n* = 10): distilled water or 0.3% citric acid at pH 3.2, with tooth brushing. They were stored for 7 days at 23 °C in an incubator, with the solutions refreshed twice a day, at 11:00 A.M. and 7:00 P.M. After storage, the specimens were rinsed for 10 min and then subjected to simulated brushing (V8, JWE GmbH, Eschborn, Germany). Each specimen was mounted, and the chamber was filled with a 1:3 toothpaste-distilled water slurry in accordance with the ISO 11609:2010 standard [30]. The toothpaste (Colgate Total, Colgate-Palmolive Arabia Ltd., Dammam, Saudi Arabia) has a relative dentin abrasiveness (RDA) of 70, which is considered low to moderate. An adequate level of toothpaste-distilled water slurry was maintained in the chamber throughout the brushing period. The specimen was brushed using toothbrushes (Oral-B, Procter & Gamble, Cincinnati, OH, USA) with a vertical load of 2N and a linear back-and-forth motion of 10 mm at 1.5 Hz. A total of 3650 cycles, equivalent to 7300 strokes, was applied to approximate roughly 1 year of intraoral use, assuming approximately 20 brushing strokes per tooth surface per day [31].

### 2.5. Gloss Measurement

Gloss was measured with a glossmeter (Novo-Cure, Rhopoint Instruments, East Sussex, UK) set to a 60° geometry, the standard geometry for dental materials evaluation, and a 2 × 2 mm^2^ measurement area. The device was calibrated after each session against the manufacturer’s tile, where 0 GU marks the low end and 1000 GU the high end. To maintain identical specimen placement, a simple jig was used to center the specimen over the aperture, and an opaque cover was added to prevent stray room light from affecting the readings. After each reading, the specimen was rotated 90° around its center to capture any directional effects in the finish. Four readings were taken per specimen at baseline and after storage, combined with simulated brushing. The four values were then averaged to report the specimen’s gloss (GU).

### 2.6. Surface Roughness Measurement

Surface roughness was measured using a high-resolution non-contact optical profilometer (Contour GT-K, Bruker Corporation, Billerica, MA, USA). For each specimen, four readings were taken at evenly spaced spots to capture the arithmetic mean roughness (Ra, in µm). The specimen was processed with a Gaussian S-filter with a cutoff of 0.8 mm before measurement. The scan was performed vertically using a 5× objective and a 1 mm × 1 mm field of view, which is adequate to capture the texture without being too wide. The profilometer was calibrated before each group’s testing, following the manufacturer’s recommendations.

The gloss and surface roughness were measured after surface treatment (stained or polished) (referred to as baseline values) and after storage combined with simulated brushing. The gloss and surface roughness measurements were taken by a single person to minimize variation in measurements.

### 2.7. Scanning Electron Microscopy Imaging

The specimens were imaged after surface treatment (baseline) and after combined storage and simulated tooth brushing using a scanning electron microscope (JSM-6610LV, JEOL, Tokyo, Japan). Before imaging, the samples were prepared by sputter-coating with gold. The SEM micrographs at 1000× magnification were then acquired with a working distance of 10 mm and an accelerating voltage of 10 kV. Figure 2 presents the flow chart detailing the study process.

### 2.8. Statistical Analysis

All statistical analyses were performed using R software (version 4.x; R Foundation for Statistical Computing, Vienna, Austria). Normality assumptions were assessed using the Shapiro–Wilk test, with *p* > 0.05 indicating normality. Data are summarized as mean ± standard deviation (SD). One-way ANOVA and Welch’s pairwise *t*-tests with Holm’s correction were used to assess differences among material groups and subgroups. The main effects and interactions of material, surface treatment, and storage medium on the changes in gloss and surface roughness were evaluated using HC3-robust Type III factorial models. Statistical significance was set at α = 0.05.

## 3. Results

### 3.1. Gloss (GU)

Table 2 presents the mean ± SD of gloss values at baseline and after storage/brushing. The mean difference between the measurements indicated a significant reduction in gloss for all materials, irrespective of the surface treatment or storage/brushing (*p* < 0.05).

Pairwise comparisons (Table 3) revealed that for stained specimens subjected to acid storage/brushing, LU exhibited significantly higher gloss than ONX (*p* = 0.003) and T2 (*p* < 0.001). In comparison, ONX also showed significantly higher gloss than T2 (*p* = 0.002). For stained specimens subjected to water storage/brushing, LU showed significantly higher gloss than T2 (*p* = 0.008), whereas the difference between ONX and LU was not significant (*p* > 0.05). ONX demonstrated significantly higher gloss than T2 (*p* < 0.001). Regarding polished specimens subjected to acid storage/brushing, ONX exhibited significantly higher gloss than both T2 (*p* = 0.007) and LU (*p* < 0.001), whereas the difference between T2 and LU was not significant (*p* = 0.07). For polished specimens subjected to water storage/brushing, ONX exhibited significantly higher gloss than both T2 and LU (*p* < 0.001), whereas the difference between T2 and LU was not significant (*p* = 0.07).

The comparison of gloss of materials between acid and water storage/brushing was not significant for both stained and polished specimens (*p* > 0.05) (Table 3).

One-way ANOVA (Table 4) revealed a significant material effect within each surface treatment (stained or polished) and storage (acid/water)/brushing (*p* < 0.001), indicating significant differences in gloss among the tested materials.

Type III factorial analysis demonstrated significant main effects of material and surface treatment, as well as a significant surface-treatment × storage/brushing interaction (*p* < 0.05). However, the main effect of storage/brushing, the material × storage/brushing interaction, and the three-way interaction were not significant (*p* > 0.05) (Table 5).

### 3.2. Surface Roughness (R_a_)

Table 6 presents the mean ± SD of the R_a_ measurements at baseline and after storage/brushing. R_a_ generally increased significantly after storage/brushing across the study groups (*p* < 0.05). However, the increase was not statistically significant for T2 specimens with a polished surface subjected to acid storage/brushing (*p* = 0.11).

Pairwise comparisons (Table 7) revealed that, among stained specimens subjected to acid storage/brushing, both T2 and ONX were significantly rougher than LU (*p* < 0.001). In contrast, the difference between T2 and ONX was not significant (*p* = 0.21). Among stained specimens subjected to water storage/brushing, T2 showed significantly higher surface roughness than both LU and ONX (*p* < 0.001). In comparison, no significant difference was observed between ONX and LU (*p* > 0.05). Regarding polished specimens subjected to either acid or water storage/brushing, no significant difference was observed between the materials (*p* > 0.05).

Acid and water storage/brushing produced comparable roughness values for the polished specimens. However, among stained specimens, significant differences between acid and water storage were observed for T2 and ONX, whereas LU showed no significant difference between the two storage conditions (Table 7).

One-way ANOVA (Table 8) revealed significant differences in surface roughness among materials under all surface-treatment and storage/brushing conditions (*p* < 0.05). The effect was highly significant for stained specimens (*p* < 0.001) and remained significant for polished specimens under both acid (*p* = 0.007) and water (*p* = 0.003) storage/brushig.

Type III factorial tests (Table 9) demonstrated significant main effects of material, surface treatment, and storage/brushing, as well as significant two-way and three-way interactions among these factors (*p* < 0.05).

### 3.3. SEM Analysis

Figure 3, Figure 4, Figure 5 and Figure 6 present SEM images of the study materials at baseline and after storage (acid/water)/tooth brushing. At baseline, T2 (Figure 3a,b and Figure 4a,b) showed mildly scattered filler particles; on the other hand, ONX (Figure 3c,d and Figure 4c,d) exhibited a more homogeneous surface, followed by LU (Figure 3e,f and Figure 4e,f). However, after the storage/brushing, all materials except LU showed substantial surface changes. T2 material exposed to acid storage/brushing (Figure 5a,b) exhibited a roughened surface and irregular flecks compared to that exposed to water (Figure 6a,b). ONX, in particular, when subjected to storage/brushing (Figure 5c,d and Figure 6c,d), showed substantial surface alterations (irregular flecks, increased roughness, and surface defects consistent with chemo-mechanical degradation) compared to the baseline and other materials. In contrast, LU subjected to storage/brushing showed a homogeneous surface similar to that observed at baseline (Figure 5e,f and Figure 6e,f). Thus, it was evident that ONX and T2 exhibited notable changes in their surface topography compared to their baseline surfaces.

## 4. Discussion

The present study evaluated the gloss and surface roughness of stained and polished 3D-printed materials (ONX and T2) and compared the findings with those of the well-established CAD/CAM-milled RNC material (LU) after storage/brushing. The outcome of the study showed highly significant main effects of materials, surface treatment, and equally large material and surface-treatment interactions on gloss. However, no significant effect of storage/brushing was observed. Therefore, the first null hypothesis related to gloss was rejected. In contrast, we observed significant main effects of material, surface treatment, and storage/brushing, as well as equally large interactions among the main effects. Consequently, the second null hypothesis related to surface roughness was accepted. Table 10 presents the overall findings of the study.

The post-storage and tooth-brushing results demonstrated a clear influence of surface treatment on the performance of the tested materials. LU retained more gloss under stained conditions compared to ONX and T2. Under polished conditions, the 3D-printed materials showed slightly better gloss retention compared to LU. The results for surface roughness also showed a similar material-dependent pattern, with the 3D-printed materials (ONX and T2) with a polished surface having better surface properties than LU. In comparison, the stained surfaces had better properties for LU. These results show that the relative surface performance of the 3D-printed and the milled materials is influenced by the surface treatment protocol: 3D-printed materials gain a slight advantage after polishing, while the opposite is true after staining.

Sarıcı and Dayı reported that surface-treatment procedures like staining and polishing had a significant effect on surface characteristics and resistance of restorative materials [32]. Staining and storage/mechanical aging have also been reported to have less desirable surface properties for some 3D-printed materials. This behavior could be related to the differences in filler content and distribution, degree of polymer conversion, and characteristics of polymerization between 3D-printed and conventionally processed resin-based materials. These differences can make it more susceptible to degradation of the surface during staining and mechanical abrasion, leading to higher surface roughness and loss of gloss. The results of the present study do, however, show that this effect is material- and treatment-specific, in that the 3D-printed materials possessed desirable surface attributes when polished [33,34,35].

Our finding that polished ONX demonstrated comparatively better surface performance than LU following storage/brushing is consistent with previous studies reporting favorable surface characteristics of appropriately post-polymerized 3D-printed resin materials under mechanical and abrasive challenges [36,37]. Surface characterizations of post-polymerized 3D-printed and high-performance resin materials indicate that, with appropriate polymerization and surface treatment, gloss retention, surface integrity, and resistance to mechanical degradation can be enhanced [37,38]. Polishing may also be used to further reduce surface irregularities, resulting in a smoother surface profile and thus leading to better gloss and surface roughness [39].

In contrast, the surface characteristics of RNC materials such as LU may be influenced by their heterogeneous resin–filler structure and the exposure or displacement of filler particles during mechanical abrasion, which can contribute to changes in gloss and surface topography [2,29,34,37]. Thus, the higher performance of polished ONX in the present study might be attributed to the difference in material composition, the difference in polymerization characteristics, the response of the material to the polishing protocol, or a combination of these factors.

The relative advantage exhibited by the polished 3D-printed materials was not observed in the stained materials, which showed that LU had better gloss retention and surface characteristics, especially after the acid storage. The favorable performance of LU may be partly related to its high inorganic filler content, which may contribute to greater resistance to surface degradation under chemical and mechanical challenges. Previous in vitro studies have similarly suggested favorable resistance of LU to acid-induced surface degradation and mechanical abrasion [40,41]. Thus, the increase in filler content in LU can be regarded as a possible contribution to this enhanced ability to retain gloss after staining and acid storage/brushing, and should not be interpreted as a clear cause.

Comparing the 3D-printed materials in both polished and stained specimens, particularly following acid storage, T2 exhibited a greater reduction in gloss than ONX. The results show that the two 3D-printed materials exhibited different responses to the chemical and mechanical stress, even though both were made by the same manufacturer. Because the detailed compositions of T2 and ONX are proprietary, particularly for T2, the specific material factors responsible for this difference cannot be established from the present study. Differences in the resistance to acid exposure or mechanical abrasion may be attributed to variations in resin chemistry, filler characteristics, degree of polymer conversion, or polymer network structure [42,43].

These findings, taken together, are consistent with those of a previous in vitro study comparing 3D-printed resins, which reported that baseline translucency and gloss were highest for LU, followed by T2 and ONX, aligning with superior polish and optical properties of polished 3D-printed materials. However, after acid storage/brushing, LU outperformed the 3D-printed materials, with ONX performing better than T2. This observation suggests that while 3D-printed restorations are advantageous when polished, they are less resistant in stained, acid storage/brushed conditions [27].

Surface roughness is an important consideration for restorative materials because increased surface irregularities may promote plaque and pigment retention. A R_a_ threshold of ≥0.2 µm has been related to increased bacteria retention [22,23]. In the present study, the surface roughness response differed according to both material and surface treatment. Overall, the polished 3D-printed materials in general had lower or similar roughness as LU after storage/brushing. The favorable surface characteristics of the polished 3D-printed materials may be related to the ability of polishing to reduce surface irregularities and produce a relatively smooth surface profile. This is in accordance with a previous study which has reported favorable surface characteristics for appropriately post-polymerized and polished 3D-printed resin materials [44]. In contrast, following staining, LU demonstrated significantly lower roughness than the 3D-printed materials, particularly after acid storage/brushing. The application of a staining or glaze layer introduces an additional surface interface that may be susceptible to chemical and mechanical degradation. Consistent with the current study, previous studies have reported changes in surface topography and integrity of resin-based restorative materials after staining and subsequent mechanical aging [12,45]. With the available information and proprietary composition, the increased roughness of ONX and T2 can thus be attributed to degradation of the surface being stained. However, cracking, delamination, and detachment of the stained layer were not directly assessed.

The different responses of the 3D-printed materials may also be related to differences in their manufacturing and polymerization characteristics. Unlike conventional manufacturing processes, in which material properties are determined by the raw material, the mechanical and surface properties of printed materials may be influenced by differences in interlayer polymerization and bonding [46,47]. Interlayer regions have been proposed as more vulnerable to water sorption and hydrolytic degradation in the absence of complete polymerization or interlayer bonding. Accordingly, an important consideration is the printing parameters. T2 was fabricated using a 100-µm layer thickness, whereas ONX was printed at 50 µm according to the respective manufacturers’ recommended protocols. The thicker layer thickness used for T2 may result in more pronounced layer-to-layer topography and greater surface irregularity, which could become more evident after staining and storage/tooth brushing. In contrast, the thinner 50-µm layers used for ONX may provide finer surface resolution and reduced layer-related topographical irregularities. These parameters may be related to surface degradation with combined exposure to acid and mechanical brushing [48,49]. The greater surface stability of LU under stained conditions may be partly associated with its high inorganic filler content of approximately 80 wt% and its CAD/CAM manufacturing process [27]. A high inorganic content may contribute to greater resistance to mechanical wear and chemical surface degradation, while the industrial processing of CAD/CAM materials may provide a relatively consistent material structure [50,51]. It has been reported that materials with higher filler concentration and better reinforcement are more resistant to erosive and abrasive challenges [27,52]. Therefore, while 3D-printed surfaces depend heavily on maintaining a flawless surface finish to stay smooth, milled materials rely on their intrinsic hardness and structural density to maintain their structural integrity [27,50].

In general, these differences observed may represent the interaction between manufacturing process, material-specific properties, and surface-treatment procedure. Acid exposure followed by simulated tooth brushing appeared to accentuate the differences between the materials, particularly under stained conditions, whereas polishing resulted in comparatively favorable surface characteristics for the 3D-printed materials. These findings suggest that the surface roughness of 3D-printed materials cannot be attributed solely to the additive manufacturing process, but rather depends on the specific material formulation and the method used to finish the surface. From a clinical perspective, the observed increase in Ra following staining/brushing is relevant because greater surface irregularity may favor plaque and pigment retention [22,23].

A key strength of this study was the direct comparison of two new 3D-printed RNC materials (T2 and ONX) with a well-established milled RNC material (LU), enabling evaluation of differences between additive and subtractive manufacturing approaches. The experimental variability was reduced by standardizing specimen dimensions, printing and post-curing parameters, surface treatments, storage, and brushing procedures. The clinical relevance of the investigation was further increased by the use of stained and polished surfaces and, therefore, the inclusion of commonly used surface treatment protocols for dental restorations. Furthermore, the combined effect of storage/brushing procedure enabled the effects of the chemo-mechanical challenges to be assessed simultaneously under controlled laboratory conditions.

Several limitations should, however, be acknowledged. As an in vitro study, the experimental conditions cannot fully reproduce the complex and dynamic oral environment, including fluctuations in pH and temperature, saliva, dietary exposure, masticatory forces, and biofilm formation. The 7-day storage period may not be adequate to simulate long-term chemical degradation, and the single citric acid concentration and pH used may not be representative of the range and duration of acidic challenges that would occur in in vivo environments. In addition, only two 3D-printed materials from the same manufacturer and one milled resin were evaluated, limiting the generalizability of the findings to other commercially available materials. The use of a single staining system and standardized staining protocol may similarly limit extrapolation to other stains, glazing systems, shades, and application techniques. Another limitation of this study is that thermal cycling was not included as an aging condition. Although the experimental protocol incorporated chemical aging in citric acid and mechanical aging through simulated toothbrushing, it did not reproduce the repeated temperature fluctuations encountered intraorally. A further limitation is that the thickness of the manually applied OptiGlaze layer was not directly measured. Although a standardized application protocol and a consistent application procedure were used to minimize variability among specimens, differences in coating thickness or uniformity may have contributed to variations in the measured surface roughness and gloss.

Future studies should involve more clinically representative and/or longer-term aging processes such as thermocycling, pH cycling, saliva exposure, and chewing simulation. Assessing a wider variety of 3D-printed and milled resin-based restorative materials from different companies would help to establish whether the differences are related to material properties or the manufacturing process. Furthermore, incorporation of 3D surface parameters and complementary analytical techniques, such as SEM with elemental or chemical analysis, could provide greater insight into the mechanisms underlying surface degradation and changes in surface morphology. Finally, in vivo long-term studies and clinical trials are needed to evaluate whether changes in gloss and surface roughness correspond to clinically relevant differences in esthetic and functional success of the definitive 3D-printed restorations.

## 5. Conclusions

This study evaluated the effect of surface treatment and storage/tooth brushing on the gloss and surface roughness of the restorative materials. The study results showed that both the surface treatment and the material type had a statistically significant influence on the surface characteristics, with stained samples showing higher surface degradation than the polished samples. Gloss values decreased significantly after storage/tooth brushing for all materials, while surface roughness typically increased. Among stained specimens, Lava Ultimate exhibited the most favorable surface properties, maintaining the highest gloss and lowest roughness, whereas Tough 2 experienced the greatest surface degradation. Polished specimens exhibited a relatively uniform surface roughness, with the highest gloss being obtained for ONX. While the storage medium had a less pronounced effect on gloss, significant effects and interactions between storage and brushing were observed for surface roughness, suggesting that material and surface treatment modulate this response. The outcomes highlight the importance of the material type and surface treatment when choosing restorative materials to achieve the desired surface quality and esthetic stability. Nevertheless, this study should be considered with the limitations of the in vitro protocol used. Further research involving extended thermomechanical aging, varied staining and polishing protocols, and intraoral biofilm exposure is recommended to better determine the long-term clinical performance of these materials.

## Figures and Tables

**Figure 1:**
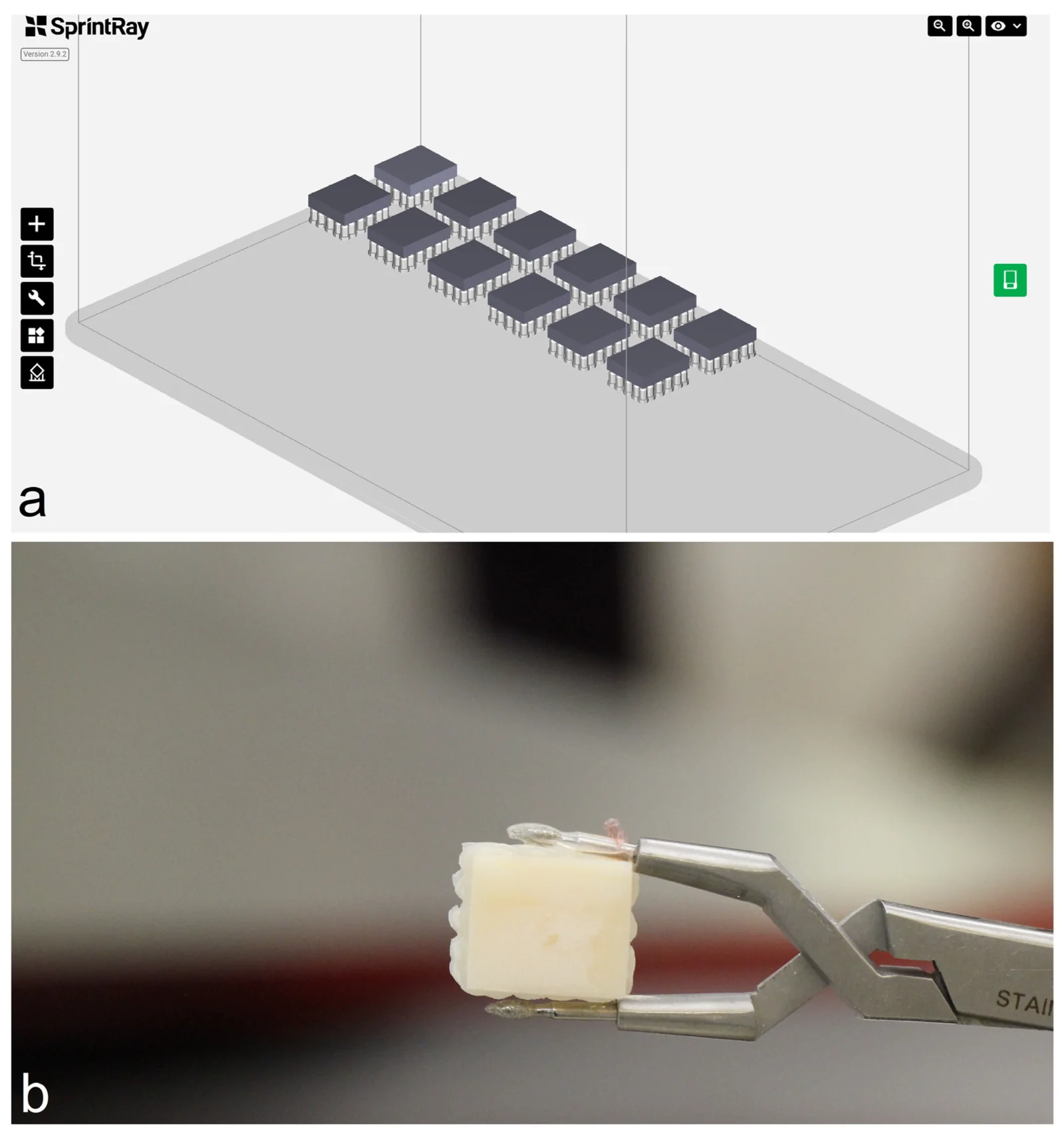
(**a**) CAD design of the specimens and (**b**) representative 3D-printed specimen.

**Figure 2:**
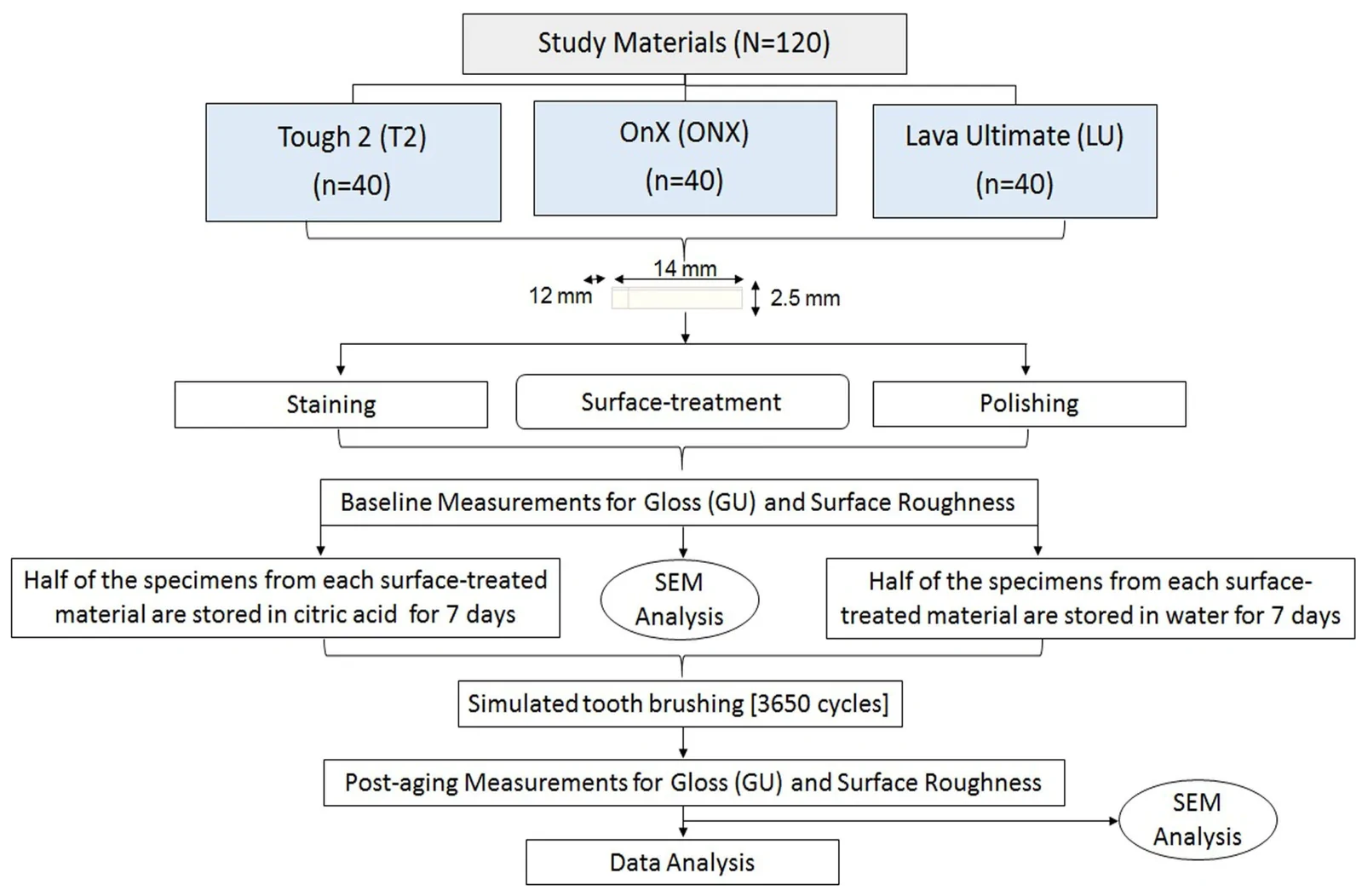
Flow chart detailing the study process.

**Figure 3:**
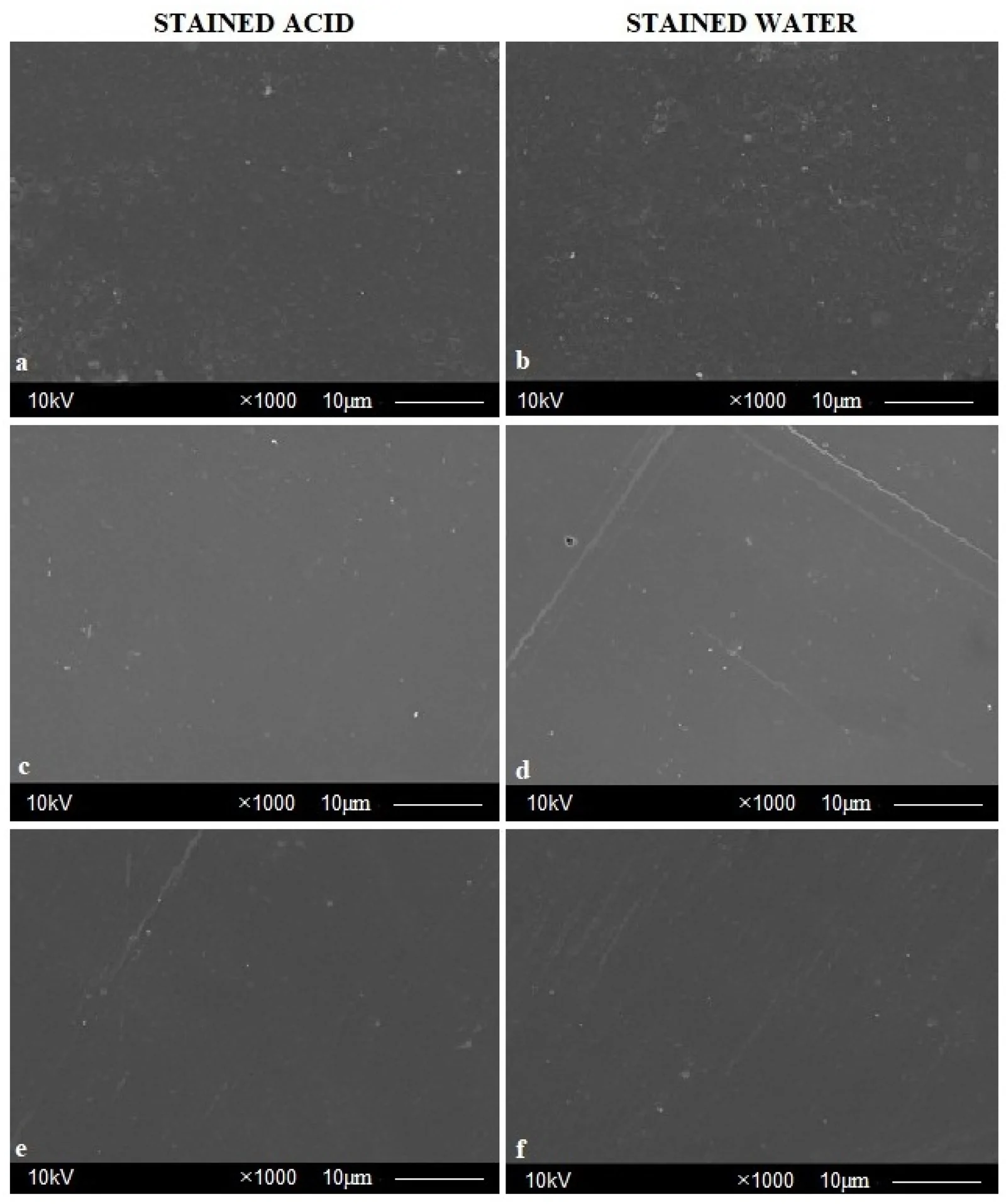
Scanning electron microscopy images (×1000 magnification) of stained specimens at baseline: T2 (**a**,**b**) T2; ONX (**c**,**d**) ONX; and LU (**e**,**f**).

**Figure 4:**
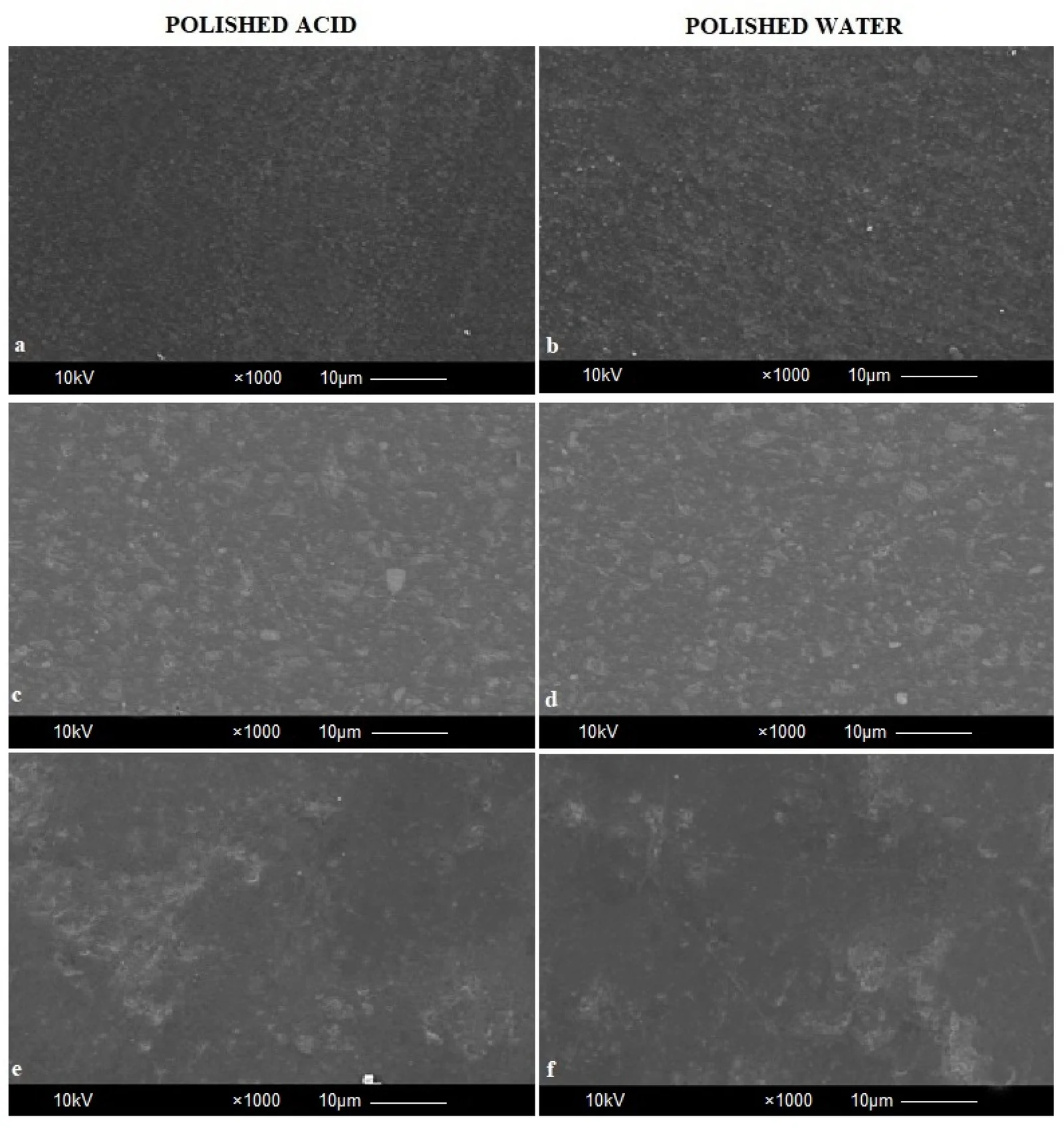
Scanning electron microscopy images (×1000 magnification) of polished specimens at baseline: T2 (**a**,**b**) T2; ONX (**c**,**d**) ONX; and LU (**e**,**f**).

**Figure 5:**
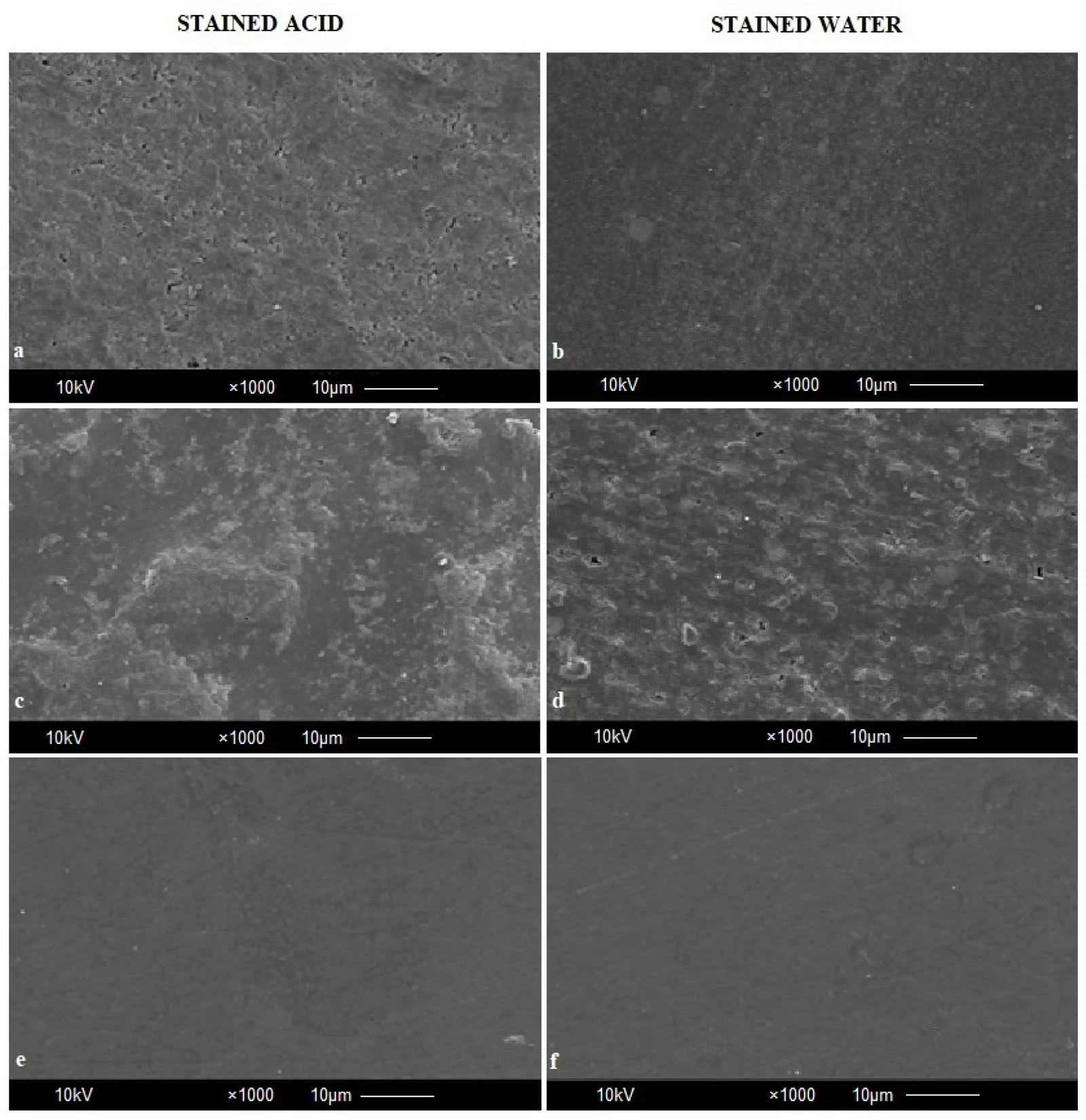
Scanning electron microscopy images (×1000 magnification) of stained specimens after storage/brushing: T2 (**a**,**b**) T2; ONX (**c**,**d**) ONX; and LU (**e**,**f**).

**Figure 6:**
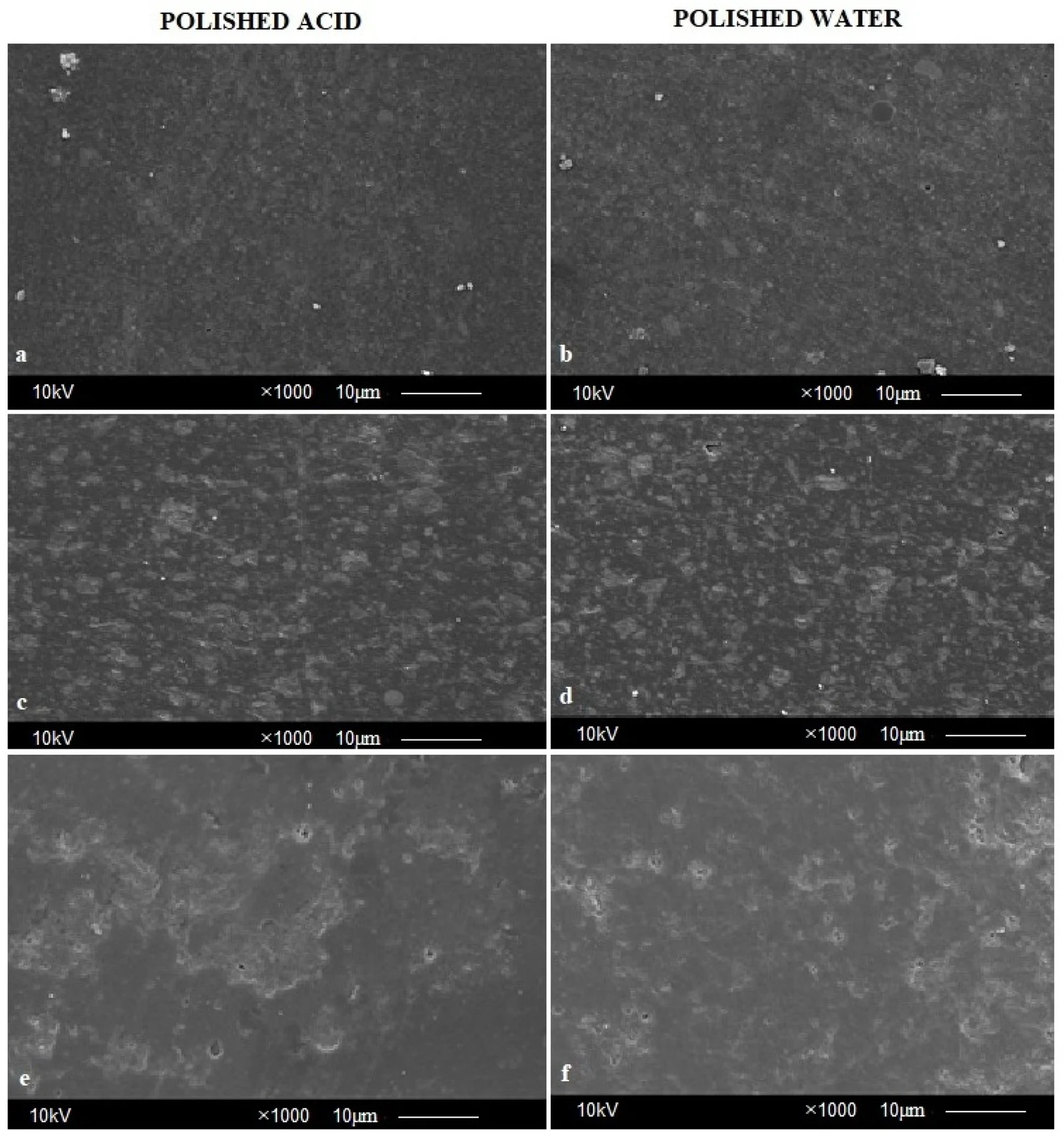
Scanning electron microscopy images (×1000 magnification) of polished specimens after storage/brushing: T2 (**a**,**b**) T2; ONX (**c**,**d**) ONX; and LU (**e**,**f**).

**Table 1:** Detailed specifications pertaining to the study materials.

Material/Resin	Shade/Acronym/Batch Number	Manufacturer Details	Composition
Tough 2/3D printing resin material	A1/T2/SRI 0202164	SprintRay, Los Ángeles, CA, USA	* Methacrylic acid esters, photo initiators, and pigments
ONX/3D printing resin material	A1/ONX/SRI 0202057	SprintRay, Los Ángeles, CA, USA	* Methacrylate monomers and oligomers, acrylic monomers, inorganic ceramic fillers, and photo initiators
Lava Ultimate (LU)/CAD/CAM milling material	A1/LU/5074A1-LT	3M ESPE, St. Paul, MN, USA	20 wt% cross-linked polymer matrix (TEGDMA), up to 80 wt% cluster nanofillers containing zirconia (4–11 nm), silica (20 nm), and aggregated zirconia or silica

* Proprietary information. TEGDMA: Triethylene glycol dimethacrylate.

**Table 2:** Mean comparison of the gloss (GU) among materials at baseline and after storage/brushing (*n* = 10).

Materials	Surface Treatment	Storage/ Brushing	Baseline	After Storage/ Brushing	Mean Difference	*p*
			Mean ± SD	Mean ± SD	Mean ± SD	
T2	Stained	Acid	190.81 ± 2.65	173.43 ± 4.46	−17.39 ± 2.05	<0.001 *
		Water	191.72 ± 3.66	176.85 ± 5.12	−14.87 ± 6.37	<0.001 *
	Polished	Acid	124.43 ± 1.42	118.12 ± 2.28	−6.31 ± 2.05	<0.001 *
		Water	124.30 ± 1.49	116.00 ± 1.49	−8.30 ± 0.91	<0.001 *
ONX	Stained	Acid	191.63 ± 5.74	180.50 ± 4.14	−11.13 ± 4.65	<0.001 *
		Water	191.96 ± 4.81	182.80 ± 3.43	−9.16 ± 5.77	<0.001 *
	Polished	Acid	124.48 ± 2.26	121.02 ± 2.01	−3.46 ± 1.60	<0.001 *
		Water	124.46 ± 1.78	120.39 ± 2.10	−4.07 ± 1.30	<0.001 *
LU	Stained	Acid	188.30 ± 2.01	186.00 ± 2.91	−2.30 ± 2.64	0.031 *
		Water	187.40 ± 3.13	185.00 ± 3.23	−2.40 ± 2.01	0.020 *
	Polished	Acid	119.87 ± 2.08	115.45 ± 3.63	−4.43 ± 3.39	<0.001 *
		Water	120.00 ± 1.89	113.80 ± 3.16	−6.20 ± 2.36	<0.001 *

* Statistically significant (*p* < 0.05).

**Table 3:** Comparison of mean gloss (GU) between the materials after storage/brushing.

Surface-Treatment	Storage/ Brushing	Materials		
		T2	ONX	LU
Stained	Acid	173.43 ± 4.46 ^a,A^	180.50 ± 4.14 ^b,A^	186.00 ± 2.91 ^c,A^
	Water	176.85 ± 5.12 ^a,A^	182.80 ± 3.43 ^b,A^	185.00 ± 3.23 ^b,A^
Polished	Acid	118.12 ± 2.28 ^a,b,A^	121.02 ± 2.01 ^b,A^	115.45 ± 3.63 ^a,A^
	Water	116.00 ± 1.49 ^a,A^	120.39 ± 2.10 ^b,A^	113.80 ± 3.16 ^a,A^

Different lowercase superscript letters (a, b, c) within a row indicate significant differences among materials (T2, ONX, and LU) (*p* < 0.05); shared lowercase letters indicate no significant differences. Shared uppercase superscript letters (A) within the same material and surface-treatment condition indicate no significant difference (*p* > 0.05).

**Table 4:** Comparison of mean gloss (GU) across materials after storage/brushing.

Surface-Treatment	Storage/ Brushing	F	df	*p*	η^2^
Stained	Acid	26.21	2, 27	* <0.001	0.66
	Water	11.03	2, 27		0.45
Polished	Acid	10.43	2, 27		0.44
	Water	20.33	2, 27		0.60

* Statistically significant (*p* < 0.05).

**Table 5:** Effect of Independent factors and interactions on the gloss (GU).

Independent Factors and Interactions	df	F	*p*
Material	2, 108	20.117	<0.001 *
Surface-treatment	1, 108	7828.414	<0.001 *
Storage/brushing	1, 108	0.006	0.940
Material × Surface-treatment	2, 108	28.003	<0.001 *
Material × Storage/brushing	2, 108	0.867	0.427
Surface-treatment × Storage/brushing	1, 108	4.516	0.039 *
Material × Surface-treatment × Storage/brushing	2, 108	0.979	0.383

* Statistically significant (*p* < 0.05).

**Table 6:** Mean comparison of surface roughness (R_a_) of the materials at baseline and after storage/brushing (*n* = 10).

Materials	Surface Treatment	Storage/ Brushing	Baseline	After Storage/ Brushing	Mean Difference	*p*
			Mean ± SD	Mean ± SD	Mean ± SD	
T2	Stained	Acid	0.17 ± 0.03	2.42 ± 0.28	2.25 ± 0.27	<0.001 *
		Water	0.17 ± 0.05	1.63 ± 0.27	1.46 ± 0.29	<0.001 *
	Polished	Acid	0.16 ± 0.02	0.18 ± 0.03	0.02 ± 0.01	0.11
		Water	0.14 ± 0.03	0.19 ± 0.05	0.05 ± 0.04	0.007 *
ONX	Stained	Acid	0.15 ± 0.04	1.76 ± 0.24	1.60 ± 0.26	<0.001 *
		Water	0.17 ± 0.04	0.66 ± 0.07	0.48 ± 0.07	<0.001 *
	Polished	Acid	0.13 ± 0.03	0.16 ± 0.02	0.03 ± 0.01	0.008 *
		Water	0.13 ± 0.03	0.18 ± 0.03	0.05 ± 0.00	0.007 *
LU	Stained	Acid	0.18 ± 0.05	0.53 ± 0.12	0.35 ± 0.15	<0.001 *
		Water	0.21 ± 0.02	0.43 ± 0.06	0.21 ± 0.05	<0.001 *
	Polished	Acid	0.13 ± 0.02	0.17 ± 0.02	0.04 ± 0.01	0.008 *
		Water	0.14 ± 0.03	0.21 ± 0.03	0.07 ± 0.03	0.009 *

* Statistically significant (*p* < 0.05).

**Table 7:** Comparison of mean surface roughness (R_a_) between the materials after storage/brushing.

Surface-Treatment	Storage/ Brushing	Materials		
		T2	ONX	LU
Stained	Acid	2.42 ± 0.28 ^a,A^	1.76 ± 0.24 ^b,A^	0.53 ± 0.12 ^c,A^
	Water	1.63 ± 0.27 ^a,B^	0.66 ± 0.07 ^b,B^	0.43 ± 0.06 ^b,A^
Polished	Acid	0.18 ± 0.03 ^a,A^	0.16 ± 0.02 ^a,A^	0.17 ± 0.02 ^a,A^
	Water	0.19 ± 0.05 ^a,A^	0.18 ± 0.03 ^a,A^	0.21 ± 0.03 ^a,A^

Different lowercase superscript letters within a row indicate significant differences among materials (T2, ONX, and LU) (*p* < 0.05); shared lowercase letters indicate no significant difference. Different uppercase superscript letters (A, B) within the same material and surface-treatment condition indicate significant differences between acid and water storage/brushing (*p* < 0.05); shared uppercase letters indicate no significant difference.

**Table 8:** Comparison of mean surface roughness (R_a_) across materials after storage/brushing.

Surface-Treatment	Storage/Brushing	F	df	*p*	η^2^
Stained	Acid	46.37	2, 27	<0.001	0.86
	Water	37.37	2, 27	<0.001	0.83
Polished	Acid	6.95	2, 27	0.007 *	0.48
	Water	8.52	2, 27	0.003 *	0.53

* Statistically significant (*p* < 0.05).

**Table 9:** Effect of independent factors and their interactions on the surface roughness (R_a_).

Independent Factors and Interactions	df	F	*p*
Material	2, 108	20.024	<0.001 *
Surface-treatment	1, 108	1167.673	<0.001 *
Storage/brushing	1, 108	6.571	0.013 *
Material × Surface-treatment	2, 108	66.433	<0.001 *
Material × Storage/brushing	2, 108	9.319	<0.001 *
Surface-treatment × Storage/brushing	1, 108	24.796	<0.001 *
Material × Surface-treatment × Storage/brushing	2, 108	5.934	0.004 *

* Statistically significant (*p* < 0.05).

**Table 10:** Overall findings of the study.

Outcome	Main Finding	Overall Interpretation
Gloss	Stained specimens showed substantially higher gloss than polished specimens. Among stained specimens, LU generally showed the highest gloss, while T2 showed the lowest. Among polished specimens, ONX showed the highest gloss.	Gloss was strongly influenced by surface treatment and material type, with a significant material × surface-treatment interaction.
Gloss after storage/ brushing	Gloss decreased significantly in all groups, but acid and water produced no significant difference.	Brushing/storage reduced gloss, whereas the storage medium had a minor additional effect.
R_a_	Roughness increased markedly after storage/brushing, particularly for stained T2 and ONX. Polished specimens remained comparatively smooth.	Surface treatment had a major influence on roughness, with significant effects of material, treatment, storage/brushing, and their interactions.
Material comparison	For stained specimens, LU maintained the lowest roughness, whereas T2 and ONX were considerably rougher. For polished specimens, no significant differences were observed among materials.	Material performance depended strongly on the surface treatment.
Overall finding	Surface treatment was the strongest determinant of both gloss and roughness, while the effect of storage medium/brushing was comparatively limited.	Polishing provided better surface smoothness, whereas staining produced higher gloss but greater susceptibility to roughness changes after storage/brushing.

## Data Availability

The original contributions presented in this study are included in the article. Further inquiries can be directed to the corresponding author.

## Abbreviations

The following abbreviations are used in this manuscript:

3D	Three-dimensional
ANOVA	Analysis of variance
CAD/CAM	Computer-aided design/computer-aided manufacturing
GU	Gloss units
LU	Lava Ultimate
ONX	SprintRay OnX
R_a_	Surface roughness
SD	Standard deviation
SEM	Scanning electron microscopy
STL	Stereolithography
T2	SprintRay Tough 2
TEGDMA	Triethylene glycol dimethacrylate
α	Alpha (significance level)
wt%	Weight percent
µm	Micrometer
